# Exploiting Thiol Modifications

**DOI:** 10.1371/journal.pbio.0020400

**Published:** 2004-11-16

**Authors:** Patricia J Kiley, Gisela Storz

## Abstract

Molecular oxygen may be necessary for life but with its beneficial properties comes formation of potentially toxic reactive oxygen species. One of the ways in which bacteria protect themselves is explained

As the premier biological electron acceptor, molecular oxygen (O_2_) serves a vital role in fundamental cellular functions, including the process of aerobic respiration. Nevertheless, with the beneficial properties of O_2_ comes the inadvertent formation of reactive oxygen species, including superoxide (O^−^_2_), hydrogen peroxide (H_2_O_2_), and hydroxyl radical (•OH); these differ from O_2_ in having one, two, and three additional electrons, respectively (Figure 1). Cells also encounter elevated levels of these reactive oxygen species when they are released by animals, plants, and insects as a defense against detrimental organisms such as microbial pathogens. Reactive oxygen species can damage cells in many ways: by inactivating proteins, damaging nucleic acids, and altering the fatty acids of lipids, which leads in turn to perturbations in membrane structure and function. The accumulation of this oxidative damage underlies the formation of many disease states in humans. It is postulated that tissue injury by these reactive oxygen species accumulates over a long period of time and plays roles in the aging process and the development of heart disease, diabetes, chronic inflammatory diseases, cancer, and several neurodegenerative diseases (Halliwell 1999).

**Figure 1:**
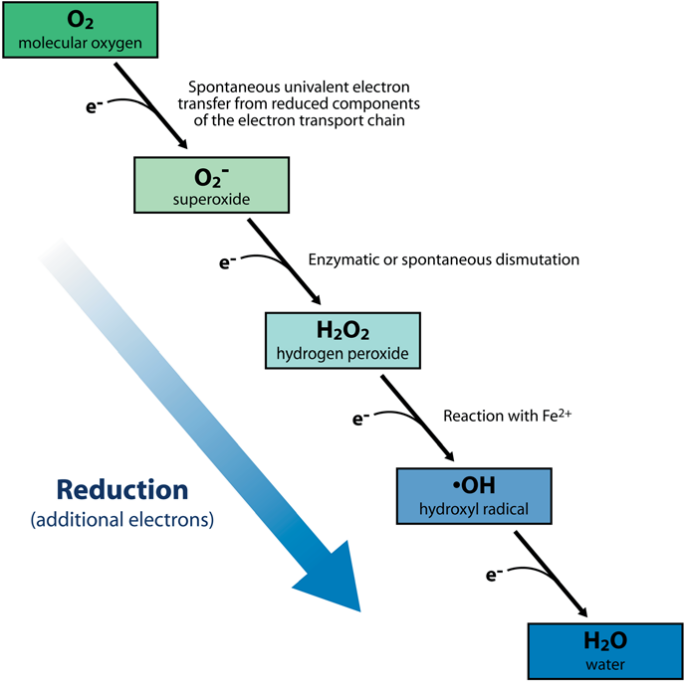
Formation of Reactive Oxygen Species The four-electron reduction of molecular O_2_ generates two molecules of H_2_O, which is O_2_ in its most reduced form. While this reduction normally occurs within the enzyme cytochrome oxidase, one-electron transfers to O_2_ also occur outside of cytochrome oxidase via inadvertent reactions with other reduced electron carriers, resulting in partially reduced and reactive forms of O_2_· H_2_O_2_ is also produced by the enzymatic or spontaneous dismutation of O_2_^−^, and •OH is generated by the reaction of iron with H_2_O_2_ (the Fenton reaction). In addition, the reactive oxygen intermediates are produced by a variety of organisms as a defense against microbial invasion. (Illustration: Rusty Howson, sososo design)

Many organisms have evolved strategies to remove reactive oxygen species and repair damage, which have enabled them to prosper from the tremendous oxidizing potential of O_2_ without succumbing to oxidative damage. Bacteria, yeast, and mammalian cells all induce the synthesis of global regulatory responses to survive oxidative insults. The consequences of oxidative stress and the corresponding defense responses have been extensively studied in Escherichia coli. For ease of study in the laboratory, the stress responses are often provoked by the external addition of chemical oxidants that specifically elevate the levels of reactive oxygen species within cells, or by the use of mutant strains that disrupt the normal “homeostatic mechanisms” for removing reactive oxygen species or the damage they do. While this primer focuses on a particular set of protective and regulatory protein modifications induced by oxidative stress in E. coli, it should be noted that many of the same mechanisms are present in other organisms; some specific examples from other species will also be described.

The major target of O_2_^−^ damage identified in bacteria is a class of dehydratase enzymes that utilize [4Fe–4S] clusters to bind their substrate (Imlay 2003; Djaman et al. 2004). Since some of these enzymes function in the citric acid cycle (also called the Krebs cycle) and in amino acid biosynthesis, high levels of O_2_^−^ lead to a requirement for certain amino acids in growth media (Imlay and is well known Fridovich 1991). H_2_O_2_ for its role in oxidizing thiol (SH) groups of cysteinyl amino acid residues in proteins. Elevated levels of H_2_O_2_ also are associated with the oxidation of other amino acids, leading to the formation of methionine sulfoxide and a variety of carbonyls. Lastly, because of its extreme reactivity, •OH targets all of the major macromolecules of cells: RNA, DNA, protein, and lipids. The extent to which membrane lipids are targets appears to depend on the presence of polyunsaturated fatty acids in lipids, which are not as prevalent in bacteria as they are in mammals.

Many enzymes that protect against oxidative damage have been identified in E. coli (Imlay 2002, 2003). Three superoxide dismutases, each of which contain a different metal center and show different expression patterns and subcellular localization, catalyze the dismutation of O_2_^−^ to H_2_O_2_. While the superoxide dismutases eliminate O_2_^−^, they also are a source of endogenously produced H_2_O_2_ in E. coli. The major enzymes involved in reducing H_2_O_2_ to H_2_O and O_2_ in E. coli are catalase and alkyl hydroperoxide reductase. There is no enzymatic mechanism for decreasing levels of •OH, produced from H_2_O_2_. Thus, levels of •OH will be directly proportional to levels of H_2_O_2_, and accordingly, catalase and alkyl hydroperoxide reductase activities are critical to oxidative stress survival.

Another component to the oxidative stress response is the reduction of oxidized thiols that arises through one of the mechanisms described below. The tripeptide glutathione and the thiol reductants glutaredoxin and thioredoxin are key to the restoration of thiols to their reduced state (SH) (Fernandes and Holmgren 2004). E. coli contains three glutaredoxins that utilize the reducing power of glutathione to catalyze the reduction of disulfide bonds (–S–S–) in the presence of NADPH and glutathione reductase. There are two thioredoxins in E. coli that also function to reduce disulfide bonds. Reduced thioredoxin is regenerated by thioredoxin reductase and NADPH. The fact that NADPH is required to maintain the reduced state of glutathione and thioredoxin indicates that the response to oxidative stress is coupled to the physiological status of core pathways that generate NADPH.

## Regulatory Roles of Thiol Modifications

**Figure 2:**
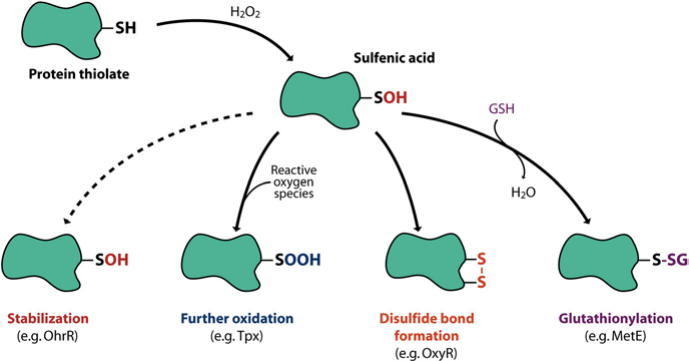
Thiol Modifications of Proteins Formation of sulfenic acid from the reaction of H_2_O_2_ with protein thiolates leads to different protein modifications, depending on the protein. In proteins without a second sulfhydryl, the sulfenic acid (–SOH) may be stabilized (e.g., OhrR) or may react with reactive oxygen species to generate the further oxidized sulfinic (–SO_2_H) (e.g., thiolperoxidase; Tpx) and sulfonic acid (–SO_3_H) derivatives. Alternatively, if a second cysteinyl residue is in proximity within the same polypeptide (e.g., OxyR) or an associated protein (e.g., Yap1 and Orp1), a disulfide bond can form between the two sulfur atoms (–S–S–). Lastly, the sulfenated cysteinyl residue can react with glutathione (GSH), leading to a mixed disulfide (e.g., MetE). (Illustration: Rusty Howson, sososo design)

As mentioned above, proteins—in particular, the thiols of cysteines—are the major targets of H_2_O_2_. The reaction of cysteinyl thiolates with H_2_O_2_ can lead to the formation of different modifications, such as sulfenic acid (–SOH), sulfinic acid (–SO_2_H), and sulfonic acid (–SO3H), as well as disulfide bond formation (–S–S–) and glutathione conjugation (–S–GSH) (Jacob et al. 2004; Poole et al. 2004) (Figure 2). These modifications often alter the structure and function of the protein. Recent progress in this field points to a common chemistry in the reaction of H_2_O_2_ with thiolates through the initial formation of sulfenic acid. In the case of proteins that have a nearby cysteinyl residue, a disulfide bond forms between the two sulfur atoms. The sulfenated cysteinyl residue also can react with a cysteinyl residue on another protein or with glutathione, leading to a mixed disulfide. If no cysteinyl residue is nearby, the sulfenated cysteine can be further oxidized to sulfinic or sulfonic acid, or it can remain in the sulfenic acid state. All but the sulfinic and sulfonic acid modifications are readily reversible by reduction, using proteins such as thioredoxin or glutaredoxin; though sulfinic acid reductase activities have recently been identified in yeast and mammalian cells (denoted sulfiredoxin and sestrin, respectively) (Biteau et al. 2003; Budanov et al. 2004).

Given the reversible nature of most forms of thiol oxidation, it has been suggested that thiol modifications can play roles in signal transduction that are similar to protein phosphorylation/dephosphorylation (Sitia and Molteni 2004). In support of this model, there are several examples of proteins whose activities are modulated by thiol oxidation and reduction.

The first of these examples is the OxyR transcription factor, which upregulates peroxide defenses in E. coli and a variety of other bacteria. OxyR contains two critical cysteines that are oxidized to form an intramolecular disulfide bond when cells encounter peroxide stress (Zheng et al. 1998; Aslund et al. 1999). Disulfide bond formation is associated with a conformational change that alters OxyR binding to DNA and allows the protein to activate the transcription of genes encoding enzymes, such as catalase and the alkylhydroperoxide reductase, that destroy H_2_O_2_. Once the H_2_O_2_ concentration is decreased, OxyR is reduced and the system is reset. The unusually reactive cysteine in OxyR that is oxidized by H_2_O_2_ to form the sulfenic acid intermediate can clearly be nitrosylated and glutathionylated in vitro (Hausladen et al. 1996; Kim et al. 2002), but the in vivo relevance of these other modifications is questionable (Mukhopadhyay et al. 2004).

Two other examples of redox-regulated proteins are the E. coli chaperone protein Hsp33 (Jakob et al. 2000) and the Streptomyces coelicolor anti-sigma factor, RsrA (Li et al. 2003; Paget and Buttner 2003; Bae et al. 2004). For these proteins, the cysteine residues, which form intramolecular disulfide bonds, are in a reduced state when coordinated to a zinc ion (Zn^2+^), and zinc is released upon oxidation of the thiols. For both proteins, oxidation and zinc release are associated with an opening of the protein structure. For Hsp33, this structural change allows for dimerization and activates its chaperone activity (Graf et al. 2004). RsrA, on the other hand, dissociates from a promoter specificity factor of RNA polymerase (an extracytoplasmic-function-type alternative sigma factor) allowing the transcription of genes that permit recovery from the stress (Li et al. 2003; Bae et al. 2004). Among the target gene products is a thioredoxin, which reduces the disulfide bonds that form within oxidized RsrA. Presumably, reduction of the disulfide restores the binding of zinc and its inhibitory association with the sigma factor. Thus, the RsrA regulatory circuit provides another example, comparable to OxyR, in which the modification of a regulatory protein thiol group can be linked to a change in the transcriptional output of genes that remediate stress.

The peroxide-sensing repressor OhrR from Xanthomonas campestris pv. phaseoli (Panmanee et al. 2002) and Bacillus subtilus (Fuangthong and Helmann 2002) can be inactivated by H_2_O_2_ or by organic peroxides (ROOH) formed by the oxidation of a variety of organic molecules in the cell or in the environment. The B. subtilis OhrR transcription regulator contains only a single cysteine that forms a relatively stable sulfenic acid upon its reaction with H_2_O_2_ or organic peroxides (Fuangthong and Helmann 2002). Oxidation of the single cysteine leads to the dissociation of OhrR from its DNA binding site and the derepression of the gene encoding an organic hydroperoxidase that eliminates the initial oxidizing insult.

In this issue, Hondorp and Matthews (2004) provide an example of a thiol modification that protects an enzyme activity during oxidative stress. Their data suggest that when cells encounter oxidative stress, a key cysteinyl residue near the active site of methionine synthase (MetE) is glutathionylated. This modification blocks access of the substrate and prevents further synthesis of methionine. This finding is significant in that it presents a mechanism to reversibly preserve the function of a protein during oxidative challenge. By glutathionylating a single cysteinyl residue, the protein is protected from further oxidation of that cysteinyl residue to the irreversible sulfinic and sulfonic acid forms. Once the stress is removed, the mixed disulfide bond will be readily reduced, and access to the substrate restored.

## Prevalence of Regulatory Thiol Modifications?

As illustrated by the examples above, an array of chemical modifications obtained by oxidizing cysteinyl residues has been exploited in combating oxidative stress. Yet it is important to note that not all cysteinyl residues of proteins are readily oxidized by oxidants such as H_2_O_2_. We do not currently understand all of the features that determine the reactivity of a particular thiol to H_2_O_2_ (Poole et al. 2004). The pKa of the thiolates clearly plays an important role, as thiolates are more reactive than their protonated counterparts. In addition, the contribution of protein environment to the stability of the oxidized products is also known to be a factor, but is not well understood. Given that many of the thiol modifications do not appear to be in equilibrium with the redox state of the cell, the features of the protein that determine the rate at which the modifications are formed are another important parameter.

The added complexity of the cysteine targets that compose part of a Zn binding site found for Hsp33 and RsrA raises questions about the function of the zinc. Perhaps Zn binding provides some additional control over the reactivity of the cysteine thiols, or perhaps the loss of the zinc facilitates conformational changes. Recently, the oxidative, stress-induced thioredoxin-2 from E. coli has also been shown to contain a H_2_O_2_-labile zinc site, although the loss of zinc does not change its reductase activity (Collet et al. 2003). Thus, the way this oxidatively labile Zn site affects thioredoxin function has yet to be established.

The extent of thiol oxidation within the cell remains another open question. The variety of modifications that arise from treatment with H_2_O_2_ and the experimental challenges associated with their detection has made it difficult to catalog all the proteins that are modified and all the types of modifications that exist. In this issue, Leichert and Jakob (2004) report a general method for detecting cellular proteins whose cysteinyl residues were modified after imposing an oxidative stress. Such an approach will greatly enhance our understanding of targets of oxidative stress. The method described by Leichert and Jakob also will be useful in detecting transient cysteine modifications.

The importance of monitoring transient changes in cysteines is highlighted by the recent finding that oxidation of the Yap1 activator of antioxidant genes in the yeast Saccharomyces cerevisiae requires a peroxidase denoted Gpx3 or Orp1 (Delaunay et al. 2002). In this case, H_2_O_2_ reacts with a cysteine in Orp1, forming an unstable sulfenic acid intermediate that then reacts with a cysteinyl residue of Yap1 to form an intermolecular disulfide. The disulfide undergoes an exchange with a second cysteine within Yap1 to form an intramolecular disulfide that locks Yap1 in a confirmation that masks the nuclear export signal (Wood et al. 2004). Thus, methods that allow the appearance of thiol modifications in cells to be monitored kinetically will greatly enhance our understanding of how cysteine residues become oxidized.

The examples mentioned here illustrate the versatile potential of thiol modifications. Given the reversibility of thiol oxidations and the wide range of structural constraints that can be imposed by the formation of a sulfenic or sulfinic acid or a disulfide bond, we predict there will be many more examples of regulation by thiol modification.

## Abbreviations

H_2_O_2_: hydrogen peroxide
O_2_: molecular oxygen
O_2_^−^: superoxide
•OH: hydroxyl radical
